# Complete Genome Sequence of the Putative Phosphonate Producer *Streptomyces* sp. Strain I6, Isolated from Indonesian Mangrove Sediment

**DOI:** 10.1128/MRA.01580-18

**Published:** 2019-01-24

**Authors:** Janina Krause, Shanti Ratnakomala, Puspita Lisdiyanti, Regina Ort-Winklbauer, Wolfgang Wohlleben, Yvonne Mast

**Affiliations:** a Department of Microbiology/Biotechnology, Interfaculty Institute of Microbiology and Infection Medicine, Faculty of Science, Eberhard Karls University of Tübingen, Tübingen, Germany; b Research Center for Biotechnology, Indonesian Institute of Sciences (LIPI), Cibinong, Indonesia; c German Center for Infection Research (DZIF), Partner Site Tübingen, Tübingen, Germany; University of Maryland School of Medicine

## Abstract

Streptomyces sp. strain I6 is a novel strain isolated from an Indonesian mangrove sediment sample. Bioinformatic analysis of the genome sequence of Streptomyces sp. I6 revealed 23 biosynthetic gene clusters. One of them encodes the synthesis of a putative phosphonate secondary metabolite, a class of underexplored natural compounds with great pharmaceutical potential.

## Abstract (precis)

Streptomyces sp. strain I6 is a novel strain isolated from an Indonesian mangrove sediment sample. Bioinformatic analysis of the genome sequence of Streptomyces sp. I6 revealed 23 biosynthetic gene clusters.

## ANNOUNCEMENT

Actinomycetes have turned out to be prolific sources for new antibiotics, as 70% of all known antibiotics were derived from actinomycetes (1). Unique habitats are suggested to be a good source for novel antimicrobial species that offer new natural compound chemistry (2). Indonesia is especially biodiverse (3) and may be host to unknown antibiotic-producing actinomycetes. Streptomyces sp. strain I6 is a novel isolate from a mangrove sediment soil sample from Tanjung Kelor Beach, Sekotong, West Lombok, Indonesia. The strain was isolated via selective medium, as described previously (4). In order to uncover its potential to produce novel natural compounds, we report here the whole-genome sequence and bioinformatic analysis of Streptomyces sp. I6.

For genome isolation, Streptomyces sp. I6 was cultivated for 2 days in 50 ml of R5 medium (5) at 30°C. For cell lysis, lysozyme (10 mg/ml; Serva) and achromopeptidase (5 mg/ml; Sigma) were added, as reported previously (6). Genomic DNA was extracted and purified using the Genomic-tip 100/G kit (catalog number 10243; Qiagen). The genomic DNA isolation procedure was carried out following the standard protocol provided by the manufacturer. For genome sequencing, a single SMRTbell template was prepared according to the Pacific Biosciences (PacBio) sample preparation protocol (7), and sequencing was performed with the PacBio RS II platform. The genome was assembled with the Hierarchical Genome Assembly Process (HGAP) v3.0 (8). HGAP data processing used PreAssembler v1 for filtering, PreAssembler v2, and AssembleUnitig v1 for assembly, BLASR v1 (9) for mapping, and Quiver v1 for consensus polishing, using only unambiguously mapped reads. HGAP3 settings were kept at the defaults, except for the genome size estimate parameter, which was set to 8.0 Mbp. Altogether, 119,346 filtered reads with an *N*_50_ value of 13,548 bp were assembled into two contigs, yielding a 7,054,598-bp draft sequence with a 6-fold coverage and an average G+C content of 72.47%. Genome annotation was performed with the NCBI Prokaryotic Genome Annotation Pipeline software tool (PGAP v4.6) (10), yielding 6,005 coding sequences (CDSs), 65 tRNAs, and 18 rRNAs. Using 16S marker genes, EzTaxon (11) identified the strain as most similar to Streptomyces spongiicola HNM0071, with 99.78% similarity (12). Using the Automatic Multi-Locus Species Tree (autoMLST) Web server (13), we found that Streptomyces sp. I6 is closely related to Streptomyces sp. strain CNT302, with an average nucleotide identity (ANI) of 94.0%.

In order to identify biosynthetic gene clusters (BGCs), the genome sequence was analyzed with antiSMASH version 4.0 (14), which predicted 23 BGCs. For five of them, antiSMASH predicted 100% similarity to the BGCs for tirandamycin (15), isorenieratene (16), desferrioxamine B (17), scabichelin (18), and staurosporine (19). One BGC showed 88% similarity to the echinomycin (20) BGC. The remaining BGCs were predicted to encode two terpenes, two thiopeptides, two bacteriocins, one polyketide, one linaridin, one melanin, one polyketide-siderophore hybrid, one phosphonate-nonribosomal peptide hybrid, one polyketide-lanthipeptide-polyketide hybrid, one butyrolactone-polyketide-nonribosomal peptide hybrid, and three other secondary metabolites.

A particularly interesting BGC from Streptomyces sp. I6 is the hybrid phosphonate-nonribosomal peptide cluster, which may encode a phosphonopeptide. Phosphonates in general are promising secondary metabolites due to their unique chemical properties and broad spectrum of activities (21). The key enzyme in phosphonate biosynthesis is the phosphoenolpyruvate mutase (PepM), which catalyzes the conversion of phosphoenolpyruvate to phosphonopyruvate (22, 23). A putative *pepM* gene (*ctg1_4282*) is present within the phosphonate-nonribosomal peptide BGC of Streptomyces sp. I6. This indicates that Streptomyces sp. I6 has the genetic potential to produce a phosphonopeptidic secondary metabolite.

### Data availability.

This whole-genome shotgun project has been deposited at DDBJ/ENA/GenBank under the accession number RHDP00000000. The version described in this paper is version RHDP01000000. Raw sequencing data are available under BioProject accession number PRJNA498008 and SRA accession number SRX4939820. For all software analyses, default settings were used.
